# Evaluation of CHK1 activation in vulvar squamous cell carcinoma and its potential as a therapeutic target in vitro

**DOI:** 10.1002/cam4.1638

**Published:** 2018-07-02

**Authors:** Zhihui Wang, Mette S. Førsund, Claes G. Trope, Jahn M. Nesland, Ruth Holm, Ana Slipicevic

**Affiliations:** ^1^ Department of Pathology The Norwegian Radium Hospital Oslo University Hospital Oslo Norway; ^2^ Department of Obstetrics and Gynecology The Norwegian Radium Hospital Oslo University Hospital and University of Oslo Oslo Norway; ^3^ Department of Pathology The Norwegian Radium Hospital Oslo University Hospital and University of Oslo Oslo Norway

**Keywords:** Checkpoint kinase 1, Chk1 inhibitor, immunohistochemistry, prognosis, siRNA, vulvar squamous cell carcinomas

## Abstract

CHK1 is an important regulator of the cell cycle and DNA damage response, and its altered expression has been identified in various tumors. Chk1 inhibitors are currently being evaluated as monotherapy and as potentiators of chemotherapy in clinical settings. However, to our knowledge, no previous study has investigated either the activation status or the therapeutic potential of CHK1 targeting in vulvar cancer. Therefore, we examined the expression status of activated CHK1 forms pCHK1^Ser345^, pCHK1^Ser317^, pCHK1^Ser296^, and pCHK1^Ser280^ in 294 vulvar squamous cell carcinomas (VSCC) using immunohistochemistry and analyzed their relationships with various clinicopathological variables and clinical outcome. To aid translation of preclinical studies, we also assessed cell sensitivity to the Chk1 inhibition in two vulvar cancer cell lines. Compared to the levels of pCHK1^Ser345^, pCHK1^Ser317^, pCHK1^Ser296^, and pCHK1^Ser280^ in normal vulvar squamous epithelium, high nuclear pCHK1^Ser345^ expression was found in 57% of vulvar carcinomas, whereas low nuclear pCHK1^Ser317^, pCHK1^Ser296^, and pCHK1^Ser280^ expressions were observed in 58%, 64%, and 40% of the cases, respectively. Low levels of pCHK1^Ser317^ and pCHK1^Ser280^ in the nucleus correlated significantly with advanced tumor behaviors and aggressive features. None of pCHK1^Ser345^, pCHK1^Ser317^, pCHK1^Ser296^, and pCHK1^Ser280^ forms were identified as prognostic factors. *In vitro* inhibition of CHK1 by small molecular inhibitors or siRNA reduced viability by inducing DNA damage and apoptosis of vulvar cancer cell lines. In summary, we conclude that cellular functions regulated by CHK1 are phosphorylation/localization‐dependent and deregulation of CHK1 function occurs in VSCC and might contribute to tumorigenesis. Targeting CHK1 might represent as a useful antitumor strategy for the subgroup of VSCC harboring p53 mutations.

## 1 INTRODUCTION

Vulvar carcinoma is a rare genital cancer counting for 3‐5% of all gynecological carcinomas and with an incidence ranging from 1 to 2 per 100 000 person‐years worldwide.^1^, ^2^ In Norway, the overall incidence of VSCC has increased from 1.70 to 4.66 per 100 000 person‐years in past 50 years.^3^ Radical surgery has been the standard treatment for most patients but is accompanied by physical and psychological adverse effects and a considerable morbidity.^1^, ^4^ Less radical treatments have been introduced in the last two decades, yet with no significant improvements in survival.^5^ Therefore, identification of new biomarkers, those can predict tumor behavior, could be important for the development of better treatment strategies.

Checkpoint kinase 1 (CHK1) is a central component in the ATR/CHK1/CDC25C DNA damage response pathway regulating G2/M cell cycle checkpoint.^6^ In response to DNA damage, ATR phosphorylates CHK1 at Ser^345^ (pCHK1^Ser345^) and Ser^317^ (pCHK1^Ser317^), which further promotes autophosphorylation at Ser^296^ (pCHK1^Ser296^).^7^, ^8^ Activation of CHK1 leads to phosphorylation of CDC25C at Ser^216^ (pCDC25C^Ser216^).^9^ The phosphorylated CDC25C is sequestered in the cytoplasm by 14‐3‐3 proteins, which further prevents dephosphorylation of CDK1/Cyclin B1 complex by CDC25C and its activation leading to G2/M arrest.^10^, ^11^ In addition, phosphorylated CHK1 also activates Wee1 resulting in further reduction in the CDK1/Cyclin B1 activity.^12^ During the G0/G1 transition, CHK1 can also be phosphorylated at Ser^280^ (pCHK1^Ser280^) by p90 RSK/MAPK pathway which promotes its cytoplasmic to nuclear translocation necessary for monitoring of genomic integrity.^13^^.^

Alteration of expression and activation of CHK1 has been identified in a variety of cancer types, including glioblastoma,^14^ sarcoma,^15^ breast,^16^ and colorectal cancer.^17^ In breast cancer, abnormal expression of CHK1 correlated with advanced tumor behavior.^16^ However, only few reports have studied activation and phosphorylation status of CHK1 with main focus on pCHK1^Ser345^.^15^, ^17^

Given the crucial role CHK1 plays at cell cycle checkpoint and the fact that cancer cells rely mainly on ATR/CHK1/CDC25C pathway to maintain genomic integrity due to dysfunctional ATM/CHK2/p53 pathway, therapeutic targeting of CHK1 has been investigated in the clinical trials both as a single agent and in combination with chemotherapy or radiotherapy.^18^ In some cancer types, preferential killing of p53‐deficient cells has been reported following CHK1 inhibition.^19^ To our knowledge, neither the activation status nor the therapeutic potential of CHK1 targeting has been studied in vulvar cancer previously. Thus, we investigated the expression status of pCHK1^Ser345^, pCHK1^Ser317^, pCHK1^Ser296^, and pCHK1^Ser280^ forms in a large cohort of primary vulvar squamous cell carcinomas (VSCC) and elucidated their relationships with various clinicopathological variables and clinical outcome. Furthermore, as p53 mutations have previously been reported in 44%^20^ to 90%^21^ of vulvar cancers, we also evaluated effects of the CHK1 targeting in p53 mutant vulvar cancer cell lines in vitro.

## 2 MATERIALS AND METHODS

### 2.1 Patients

A retrospective study was performed on a cohort of 294 patients with VSCC between 1977 and 2006. All patients had undergone surgery at The Norwegian Radium Hospital. The median age of patients at diagnosis was 74 years (range, 35‐96 years). Before surgery, six patients were treated with radiotherapy and three with radiotherapy/chemotherapy. Radical vulvectomy was performed on 195 (66%) patients, whereas 99 (34%) patients were subjected to nonradical surgery. Postoperative therapy has been given to 69 patients including chemotherapy in 3 patients, irradiation in 62, and irradiation/chemotherapy in 4 cases. All the patients were followed until death occurred or 5 years after study inclusion. Ninety‐nine (34%) patients died of vulvar cancer within 5 years after inclusion. The tumor stage examination was performed according to the 2009 International Federation of Gynaecology and the Obstetrics (FIGO) classification system.^22^ Histological re‐examination was performed by the coauthors (J.M.N) according to World Health Organization recommendations.^23^ Two hundred and seventy‐seven (94%) tumors were keratinizing/nonkeratinizing, 13 (5%) were basaloid, and 4 (1%) were veruccoid. Ten normal vulvar samples from patients operated for benign gynecological diseases were included as controls.

The Regional Committee for Medical Research Ethics South of Norway (S‐06012), The Data Inspectorate (04/01043), and The Social and Health Directorate (04/2639 and 06/1478) approved this study. There has been used paraffin‐embedded tumor tissue from patients with vulvar cancer diagnosed between 1977 and 2006. As all of these patients are either dead or very old, we have not been able to obtain patient consent. Permission has been obtained from The Social and Health Directorate (04/2639) to perform this study without patient consent, which applies to all participants.

### 2.2 Immunohistochemistry and statistics

Sections for immunohistochemistry were stained using the Dako EnVision^™^+ system (K8012; Dako Cooperation, CA, USA) and DAKO Autostainer. Deparaffinization, rehydration, and target retrieval were performed in a Dako PT‐link and EnVision^™^ Flex target retrieval solution with low pH. To block endogenous peroxidase, the sections were treated with Dako blocking reagent for 5 minutes. The sections were incubated with polyclonal rabbit antibodies against pCHK1^Ser345^ (LS‐C117319, 1:500, 2 μg IgG/mL, overnight at 4°C, Lifespan Biosciences Inc., Seattle, WA, USA), pCHK1^Ser317^ (IHC‐00068, 1:500, 0.5 μg IgG/mL, overnight at 4°C; Bethyl laboratories, Montgomery, AL, USA), pCHK1^Ser296^ (C4200‐15U, 1:700, overnight at 4°C; United States Biological Inc., Swampscott, MA, USA), and CHK1^Ser280^ (C4200‐05G, 1:2100, 0.5 μg IgG/mL, 30 minutes at room temperature, United States Biological Inc). All of the sample series included normal testis as positive control. Negative controls included substitutions of primary antibodies with normal rabbit IgG at the same concentration as the primary antibodies.

Expression of the different phosphorylated forms of CHK1 was categorized on the basis of the percentage of positive tumor cells (absent, 0; <10%, 1; 10‐50%, 2; >50%, 3) and staining intensity (absent, 0; weak, 1; moderate, 2; strong, 3). Immunoreactivity in cytoplasm and nucleus was calculated separately by multiplying the scores of the staining extent and intensity of each slide, and composite scores were ranged from 0 to 9. The immunohistochemical staining was evaluated without knowledge of the patient outcome. The cutoff values for the immunostaining were based on staining pattern observed in normal vulvar epithelium. Cytoplasmic immunostaining was classified as high when score >0 for pCHK1^Ser345^ and pCHK1^Ser296^, score >6 for pCHK1^Ser317^, and score >4 for pCHK1^Ser280^ and low when score = 0 for pCHK1^Ser345^ and pCHK1^Ser296^, score ≤6 for pCHK1^Ser317^, and score ≤4 for pCHK1^Ser280^. Nuclear immunostaining was evaluated as high when score >0 for pCHK1^Ser345^, score >4 for pCHK1^Ser317^ and pCHK1^Ser280^, and score >3 for pCHK1^Ser296^ and low when score = 0 for pCHK1^Ser345^, score ≤4 for pCHK1^Ser317^ and pCHK1^Ser280^, and score ≤3 for pCHK1^Ser296^.

The associations between expression of proteins and clinicopathological parameters were evaluated by Pearson's chi‐square (χ^2^), Fisher exact test, and linear‐by‐linear association. Kaplan and Meier method was used to calculate the disease‐specific survival from the date of diagnosis to vulvar cancer‐related death. Survival rate comparison was performed by the log‐rank test. A Cox proportional hazards regression model was used for both univariate and multivariate evaluation of survival. Patients were censored after 5 years. In the multivariate analysis, a backward stepwise regression with a *P* value of .05 as the inclusion criterion was used. All analyses were performed using the SPSS 18.0 statistical software package (SPSS, Chicago, IL, USA). All analyses were two‐sided, and statistical significance was considered as *P *≤* *.05.

### 2.3 Cell lines and growth conditions

Vulvar squamous cell carcinoma cell line CAL39 (DSMZ, Braunschweig Germany) was cultured in Dulbecco's modified Eagle medium (DMEM, Gibco, LifeTechnologies, Invitrogen, Oslo, Norway) supplemented with 10% Fetal Calf Serum (Biocrom, KG, Berlin, Germany) and 2 mM L‐glutamine (Lonza, Vervieres, Belgium) at 37°C in humidified condition containing 5% CO_2_. SW954 cell line (ATCC, Manassas, VA, USA) was cultured in Lanza BioWhittaker L‐15 (Leibovitz) Medium (Lonza) supplemented with 20% fetal calf serum and 2 mmol/L L‐Glutamine at 37°C in humidified conditions without CO_2_.

### 2.4 Chemical inhibitor and viability assay

AZD7762 was purchased from Selleck Chemicals (Houston, TX, USA) and prepared as a 1 mmol/L DMSO stock. Aliquots were stored at −80°C. CAL39 and SW954 (3 × 10^3^ cells/well and 6 × 10^3^ cells/well) were seeded in 96‐well plates day before treatment with AZD7762. The viability was determined by the CellTiter‐Glo^®^ Luminescent Cell Viability Assay kit (Promega, Madison, WI, USA) as described by the manufacturer after 72 and 96 hours. CAL39 and SW954 were also exposed either to a spectrum concentrations of AZD7762 (0, 62.5, 125, 250, 500, 1000, and 2000 nmol/L) for 24 hours or to a certain concentration of AZD7762 (0 and 500 nmol/L) for 24 hours, 48 hours, and 72 hours before cells were harvested for immunoblotting analysis.

### 2.5 Small interfering RNA (siRNA) transfection

CAL39 and SW954 were plated in either 6‐well plates (1 × 10^5^ cells/well and 2 × 10^5^ cells/well) or in 96‐well plates (3 × 10^3^ cells/well and 6 × 10^3^ cells/well) 24 hours before the transfection. Transfections with siRNA targeting CHK1 (OligioID: “VHS40226” Invitrogen, LifeTechnologies) and RNAi negative control duplexes (Negative Control LOW GC, 12935‐200, Invitrogen) were performed using Lipofectamine^™^ RNAiMAX transfection reagent. Cells were harvested after 48 hours for immunoblotting analysis or assessed for viability after 3 days and 5 days.

### 2.6 Antibodies and immunoblotting analysis

Primary antibodies CHK1, pCHK1^Ser345^, pCHK1^Ser317^, CDC25C, pCDC25C^Ser216^, Caspase 3 (#9662/#9664 (even mix)), and β‐actin were purchased from Cell Signaling (Beverly, MA, USA). pCDK1^Tyr15^ (ab47594) were acquired from Abcam (Cambridge, UK). pH2A.X^Ser139^ (#05‐636) was purchased from Millipore. Wee1 (sc‐5285), p53 (sc‐126), and Cyclin A (sc‐751) were obtained from Santa Cruz Biotechnology (Dallas, Tex, USA), whereas CIP2A was brought from Novus Biologicals (Littleton, Co, USA). Cells were harvested and then lysed in ice‐cold NP‐40 lysis buffer as previously described.^24^ Protein quantification was performed to ensure even loading by Bradford (Bio‐Rad Laboratories AB, Sundbyberg, Sweden) analysis. Proteins (15 μg protein/lane) were separated on Criterion TGX10% Midi Precast Gels in Tris/Glycine Buffers (Bio‐Rad, Hercules CA, USA) at 200 V for 40 minutes, and blotted on PVDF‐membrane using Bio‐Rad Transblot Turbo system according to the manufacturer's instructions. Membranes were blocked in 5% nonfat milk in TBST (20 mmol/L Tris‐Cl, 136 mmol/L NaCl [pH 7.6], 0.1% Tween 20) at room temperature for 1 hour, before they were probed with primary antibodies at 4°C overnight with gentle agitation. Secondary HRP‐conjugated antibodies were visualized using ECL‐plus reagent (GE Healthcare, Chalfont St Gils, UK) by exposure to X‐ray films.

## 3 RESULTS

### 3.1 Expression of phosphorylated CHK1^Ser345^, CHK1^Ser317^, CHK1^Ser296^, and CHK1^Ser280^ proteins

**Figure 1:**
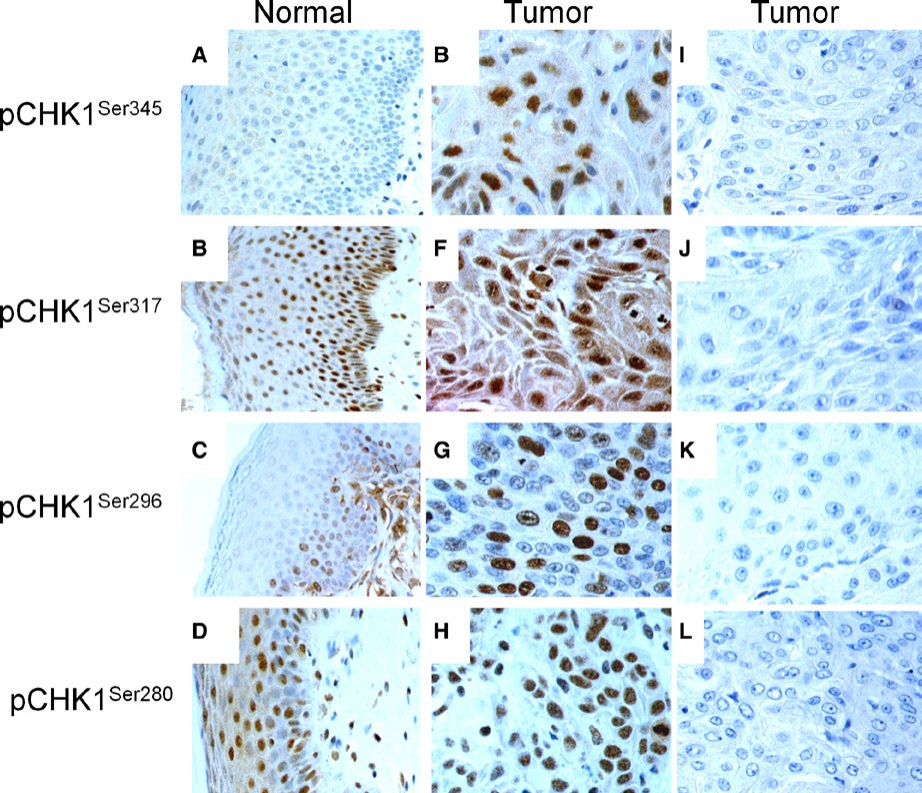
Expression of pCHK1^Ser345^, pCHK1^Ser317^, pCHK1^Ser296^, and pCHK1^Ser280^ in vulvar squamous epithelium. Immunostaining of pCHK1^Ser345^ (A), pCHK1^Ser317^ (B), pCHK1^Ser296^ (C), and pCHK1^Ser280^ (D) in normal vulvar epithelium (Magnification × 300). High expression of pCHK1^Ser345^ (E), pCHK1^Ser317^ (F), pCHK1^Ser296^ (G), and pCHK1^Ser280^ (H) and low expression of pCHK1^Ser345^ (I), pCHK1^Ser317^ (J), pCHK1^Ser296^ (K), and pCHK1^Ser280^ (L) in VSCC (Magnification × 600)

In 10 cases of normal vulvar squamous epithelium, no cytoplasmic immunoreactivity was observed for pCHK1^Ser345^ and pCHK1^Ser296^, whereas cytoplasmic staining for pCHK1^Ser317^ (score ≤6) and pCHK1^Ser280^ (score ≥6) was detected in basal, parabasal, middle, and top layers. Nuclear immunostaining was not seen for pCHK1^Ser345^, and for pCHK1^Ser296^ (score = 3), nuclear immunoreactivity was identified in basal and parabasal layer, whereas for pCHK1^Ser317^ (score ≥6) and pCHK1^Ser280^ (score ≥6), nuclear staining was detected in basal, parabasal, middle, and top layers (Figure 1A‐D).

**Figure 2:**
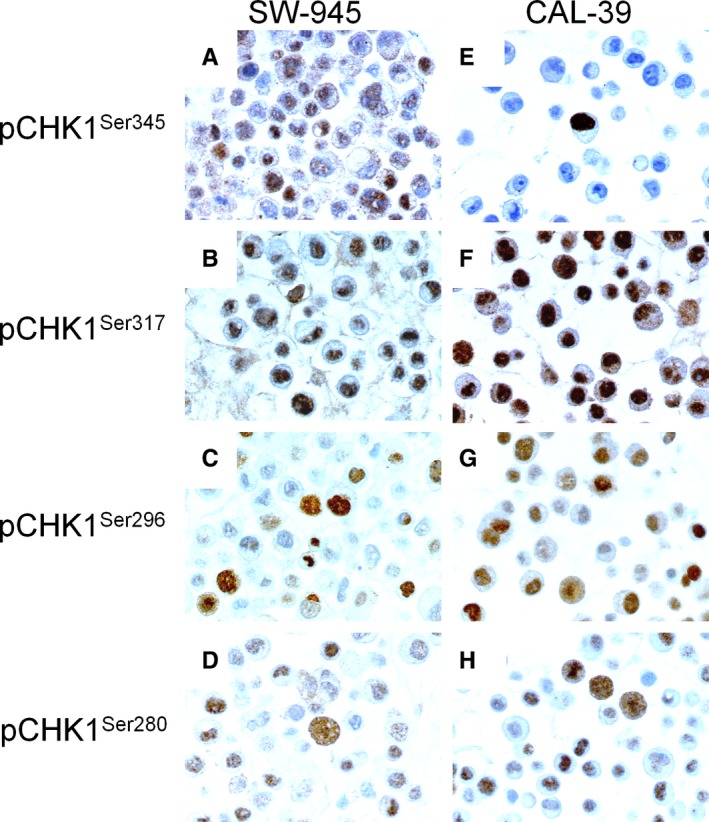
Expression of pCHK1^Ser345^, pCHK1^Ser317^, pCHK1^Ser296^, and pCHK1^Ser280^ in vulvar cancer cell lines. Immunostaining of pCHK1^Ser345^, pCHK1^Ser317^, pCHK1^Ser296^, and pCHK1^Ser280^ in SW945 (A‐D) and CAL39 (E‐H) (Magnification × 600)

In the cytoplasm, high expression of pCHK1^Ser345^ (score >0), pCHK1^Ser317^ (score >6), pCHK1^Ser296^ (score >0), and pCHK1^Ser280^ (score >4) was observed in 106 (36%), 45 (15%), 56 (19%), and 139 (47%) of the VSCC, respectively. In the nucleus, high expression of pCHK1^Ser345^ (score >0), pCHK1^Ser317^ (score >4), pCHK1^Ser296^ (score >3), and pCHK1^Ser280^ (score >4) was observed in 167 (57%), 124 (42%), 105 (36%), and 175 (60%) of the VSCC, respectively (Figure 1E‐L and Table S1). In the vulvar carcinoma cell lines, SW954 and CAL39 high levels of nuclear staining of pCHK1^Ser345^ (score >0), pCHK1^Ser317^ (score >4), pCHK1^Ser296^ (score >3), and pCHK1^Ser280^ (score >4) were observed (Figure 2).

### 3.2 Correlations between phosphorylated forms of CHK1 and G2/M cell cycle factors

As our cohort of VSCC has previously been tested for different forms of CDC25,^25^ 14‐3‐3,^26^, ^27^ CDK1 and Cyclin B1,^28^ and Wee1,^24^ we have examined the relationship between pCHK1^Ser345^, pCHK1^Ser317^, pCHK1^Ser296^, and pCHK1^Ser280^ and these factors (Tables S2 and S3). Protein levels of cytoplasmic pCHK1^Ser345^, pCHK1^Ser317^, and pCHK1^Ser280^ were positively correlated with each other. Positive correlation was also observed between nuclear pCHK1^Ser317^, pCHK1^Ser296^, and pCHK1^Ser280^ forms.

When comparing the phosphorylated CHK1 forms with different forms of 14‐3‐3, CDC25, Wee1, CDK1, and Cyclin B1, we observed that 1) in the cytoplasm as well as in the nucleus phosphorylated CHK1 forms were positively correlated with members of the 14‐3‐3 family; 2) in the nucleus, high protein levels of pCHK1^Ser345^ and pCHK1^Ser317^ were associated with high level of pCDC25C^Ser216^; 3) in the nucleus, high expression of pCHK1^Ser296^ was related to high expression of Wee1; and 4) in the nucleus, pCHK1^Ser317^ and pCHK1^Ser296^ were positively correlated with CDK1^Tyr15^ and pCyclin B1^Ser126^.

### 3.3 Association between phosphorylated forms of CHK1 and clinicopathological variables and survival

Low nuclear level of phosphorylated CHK1 proteins correlated with old age (pCHK1^Ser317^ and pCHK1^Ser296^), deeper invasion (pCHK1^Ser317^ and pCHK1^Ser280^), and large tumor diameter (pCHK1^Ser317^) (Tables S4 and S5). In addition, high cytoplasmic expression of pCHK1^Ser296^ was associated with poor histological differentiation.

**Figure 3:**
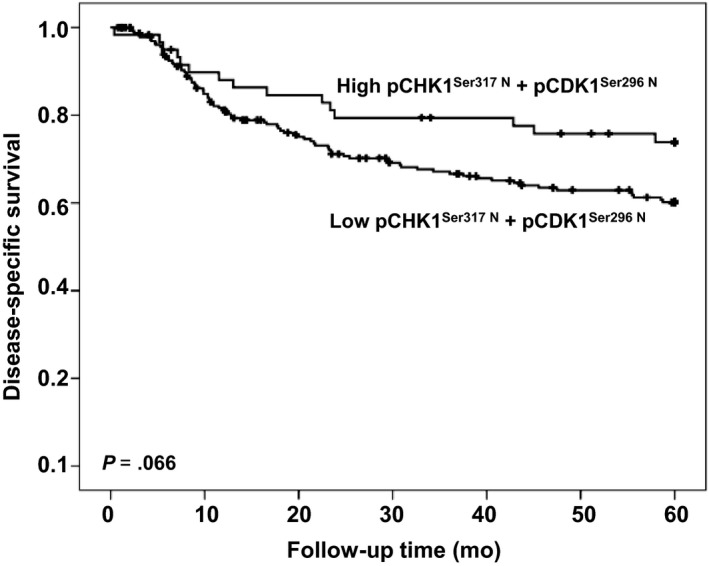
Survival curves using the Kaplan‐Meier method. The Kaplan‐Meier curves of disease‐specific survival in relation to combinations of pCHK1^Ser317 N^ + pCHK1^Ser296 N^. The *P*‐value differs slightly from the one in Table 1 due to the use of the log‐rank test as opposed to the Cox‐regression analysis

By univariate analysis, we found that neither cytoplasmic nor nuclear expression of any of the pCHK1^Ser345^, pCHK1^Ser317^, pCHK1^Ser296^, and pCHK1^Ser280^ forms was associated with disease‐specific survival. However, if samples with simultaneous low nuclear levels of pCHK1^Ser317^ and pCHK1^Ser296^ forms were analyzed as one subgroup, a tendency (P=0.066) for shorter disease‐specific survival was observed (Figure 3). None of the phosphorylated forms of CHK1 had a significant correlation with survival in either HPV‐positive or HPV‐negative group. In multivariate analysis, only lymph node metastases, age, and tumor diameter retained independent prognostic significance for patients with VSCC (Table 1).

**Table 1:** Relative risk (RR) of dying from vulvar cancer

Variables	Univariate analysis			Mutivariate analysis		
	RR	95% CI^a^	*P*	RR	95% CI	*P*
Lymph node metastases	2.54	1.97‐3.27	<.001	2.25	1.71‐2.96	<.001
Age	1.53	1.14‐2.04	.004	1.42	1.06‐2.05	.023
Tumor diameter	1.94	1.48‐2.54	<.001	1.53	1.14‐2.04	.004
Infiltration of vessel	2.28	1.50‐3.47	<.001	‐	‐	‐
pCHK1^Ser317 N^ b + pCHK1^Ser296 N^	0.60	0.35‐1.04	.069	‐	‐	‐

a 95% confidence interval.

b High vs low expression.

### 3.4 In vitro inhibition of CHK1 reduces viability of vulvar cancer cell lines

**Figure 4:**
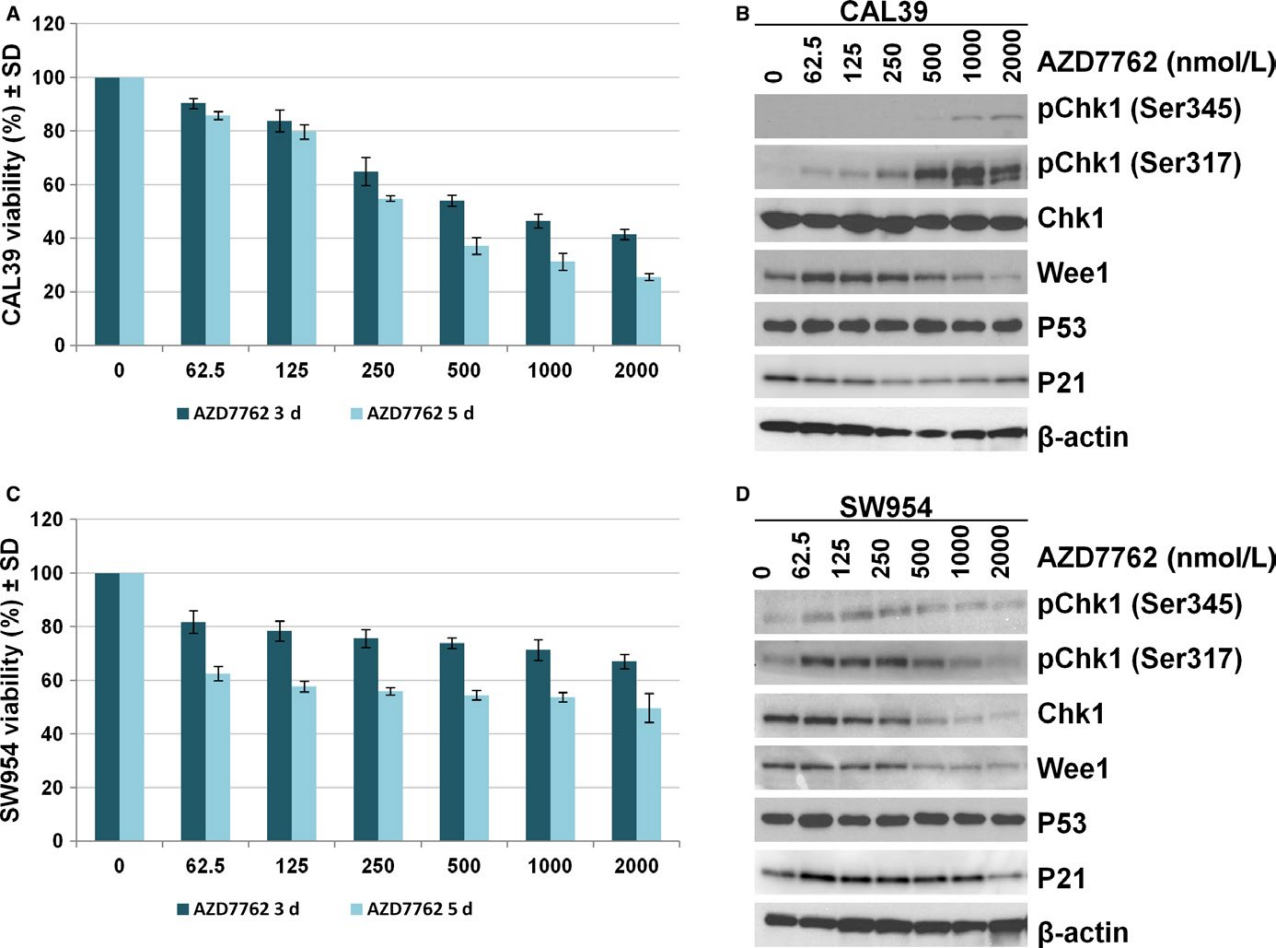
AZD7762 reduces viability in vulvar squamous cells. (A and C) Cell viability measured by CTG assay in CAL39 and SW954 exposed to the increasing concentrations of AZD7762 for 3 days and 5 days, respectively. Error bars represent the standard deviation from n = 3 independent experiments. (B and D) Immunoblotting analysis of CAL39 and SW954 cells showing increase in CHK1 phosphorylation after exposure to AZD7762 for 24 hours. β‐actin is used as loading control. Representative data from n = 3 experiments

In order to investigate the effect of CHK1 targeting in vulvar cancer, we treated CAL39 and SW954 cell lines with increasing concentrations (0.063‐2 μmol/L) of the commercially available CHK1/CHK2 inhibitor AZD7762 for up to 5 days. CAL39 cell line exhibited dose‐ and time‐dependent sensitivity to inhibitor AZD7762 (Figure 4A). In the SW954 cell line, the inhibitor was not able to reduce the viability more than 50 % even at the highest doses, suggesting that SW954 cells are more resistant to CHK1 inhibition (Figure 4C). Immunoblotting analysis showed that treatment with AZD7762 for up to 24 hours led to a dose‐dependent increase in CHK1 phosphorylation at the Ser345 and Ser317 sites in both cell lines (Figure 4B,D). However, we observed a decrease in protein level of total CHK1 in SW954 cells at doses higher than 500 nM (Figure 4B,D). Similarly, decreased protein levels of Wee1 and p53 were observed in both cell lines at higher doses (Figure 4B,D).

### 3.5 CHK1 inhibition induces DNA damage and apoptosis

**Figure 5:**
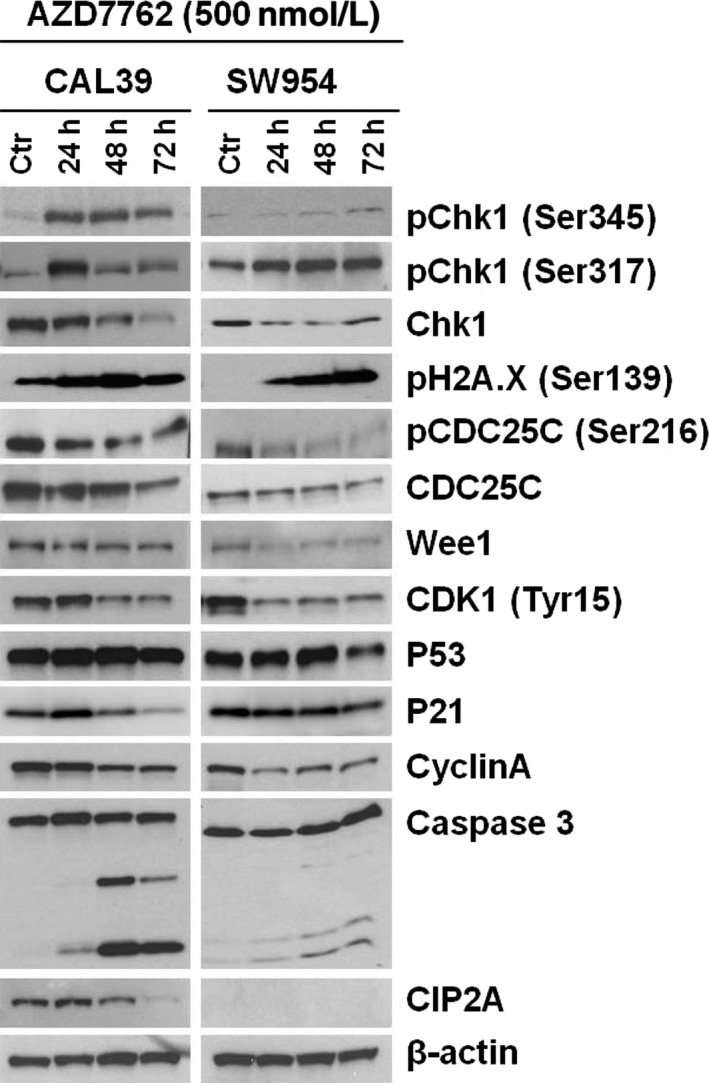
Effects of AZD7762 on protein expression of cell cycle regulators and apoptosis markers. Immunoblotting analysis of CAL39 and SW954 cells showing altered expression of proteins involved in cell cycle regulation and apoptosis after exposure to AZD7762 (500 nmol/L) for 24, 48, and 72 hours, respectively. β‐actin is used as loading control. Representative data from n = 3 experiments

To elucidate the mechanisms by which CHK1 inhibitor affect vulvar cancer cell viability, we analyzed the expression of proteins downstream of CHK1 as well as apoptotic markers after treatment with 500 nM AZD7762 for up to 72 hours. As shown by immunoblotting in Figure 5, the inhibitor led to an increase in CHK1 phosphorylation, while total levels of the protein decreased during the treatment in both cell lines. Furthermore, we observed an increase in γ‐H2A.XSer139 in both cell lines indicating an induction of the DNA damage. At the same time, the AZD7762 treatment did not lead to a significant change in p53 levels in either cell line. A slight initial upregulating followed by a downregulation of p21 was seen in CAL39 cells; no such change was observed in SW954 cells. There was a clear downregulation of cell cycle regulators CDC25C, CDK1, and Cyclin A in both lines and cleavage of caspase 3, suggesting that CHK1 inhibition affects both proliferation and apoptosis of vulvar cancer cells. Caspase 3 cleavage was more profound in CAL39 cells that display a higher sensitivity to the inhibitor. In CAL39 cells, CHK1 inhibition also led to downregulation of CIP2A, while SW954 cells showed no CIP2A expression at all.

**Figure 6:**
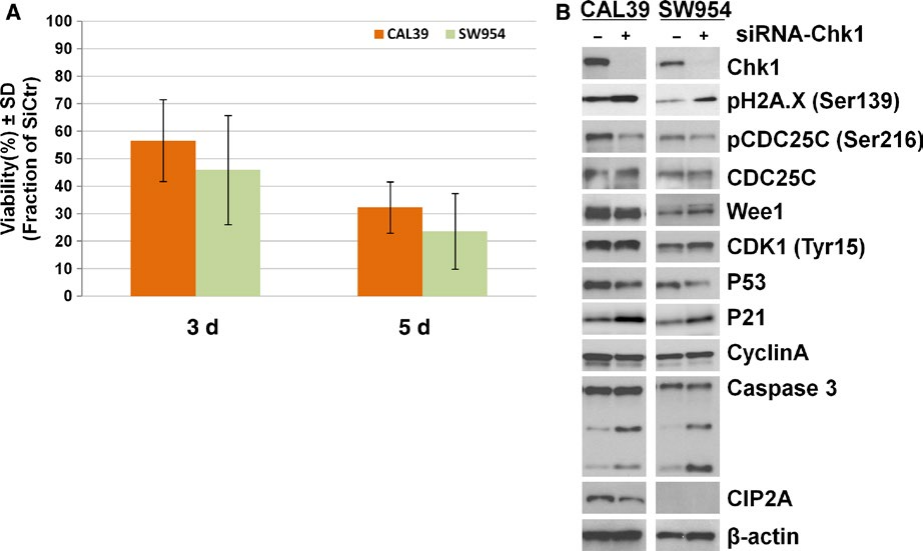
CHK1 downregulation by siRNA leads to strong reduction in viability and apoptosis. (A) Effect of siCHK1 on cell viability of CAL39 and SW954 was assessed by trypan blue cell counting 3 days and 5 days after siRNA transfection, respectively. Error bars represent the standard deviation from n = 3 independent experiments. (B) Protein levels of downstream effector proteins following siRNA transfection by immunoblotting. β‐actin was used as loading control. Representative data from n = 3 experiments

To further validate the specificity of CHK1 targeting, we transiently downregulated CHK1 in the cell lines using siRNA (Figure 6). As shown in Figure 6A, CHK1 downregulation led to a high reduction in the cell viability in both cell lines after 5 days (approximately 60% in CAL39 and 80% in SW954). Interestingly, the effect of CHK1 knockdown was stronger in the SW954 cells that displayed higher resistance to the CHK1 inhibitor previously.

The siRNA CHK1 knockdown had a less profound effect on the protein levels of the cell cycle proteins than inhibitor treatments for both cell lines (Figure 6B). However, a noticeable increase in γ‐H2A.X^Ser139^, Caspase 3 cleavage, downregulation of p53, and upregulation of p21 was observed in both lines, suggesting that a stronger apoptotic effect is induced after CHK1 siRNA knockdown (Figure 6B).

## 4 DISCUSSION

Checkpoint kinase 1 is a crucial regulator of the cell cycle and DNA damage response. In unperturbed cell cycle, CHK1 is involved in regulation of G1/S transition, S phase, as well as mitotic entry and mitosis.^29^ As a part of DNA damage response, cascade CHK1 mainly regulates G2 checkpoint activation. In current study, we have assessed activation status of CHK1 in normal vulvar squamous epithelium and cancer lesions and investigated effects of the CHK1 targeting in vitro. In normal vulvar epithelium, we found detectable expression of pCHK1^Ser317^, pCHK1^Ser296^, and pCHK1^Ser280^ forms, while no expression of pCHK1^Ser345^ was observed. Previous studies have suggested that phosphorylation of all CHK1 sites is required for efficient activation and full checkpoint proficiency in response to DNA damage.^30^ However, it is still not determined how different phosphorylation forms of CHK1 can regulate diverse aspects of genome surveillance during the cell cycle. Our results suggest that phosphorylation at Ser345 might not be required for some of these processes in normal vulvar tissue. In support of this, a study by Wlaker et al^7^ suggested that phosphorylation at Ser345 is not obligatory for CHK1 kinase activity per se, although it plays a role in CHK1 activation in the cells. Previously, pCHK1^Ser345^ has been identified in normal colonic mucosa 17 which could perhaps be related to a greater exposure of this tissue to the carcinogens, leading to elevated levels of the DNA damage and repair pathways. In normal vulvar epithelium baseline, DNA damage levels might be below the threshold required for activation of DNA damage checkpoint mediated by phosphorylation of pCHK1^Ser345^. Another interesting observation is the high expression of pCHK1^Ser280^ in normal vulvar epithelium. It has been shown that this phosphorylation can be mediated by Akt,^31^ RSK 13 in different cell types, possibly leading to cytoplasmic sequestration. Phosphorylation of this residue is not required for checkpoint function in untransformed cells.^31^ For this reason, it is likely that in normal vulvar epithelium pCHK1^Ser280^ rather functions in other aspects of the normal cell cycle progression.

Previously, altered expression of CHK1 has been reported in a variety of malignant tumors, including small cell lung cancer,^32^ breast cancer,^16^ colon cancer,^17^ glioblastoma,^14^ and sarcoma.^15^ Expression of pCHK1^Ser345^ was identified in 21% of soft tissue sarcoma,^15^ while a reduced expression of pCHK1^Ser345^ has been found in about 50% of colon cancer.^17^ Interestingly, CHK1 expression was often positively correlated with more aggressive tumors,^16^, ^33^ whereas elevated levels of pCHK1^Ser345^ correlated with increased radio‐resistance in metastatic brain and patients with lung cancer.^14^ However, in colon cancer, neither CHK1 nor pCHK1^Ser345^ were associated with Dukes stage and lymph node metastasis.^17^ In our study, we observed a high level of pCHK1^Ser345^ in the nucleus in 57% of VSCC although this expression was not associated with any clinicopathological features. It is known that activated pCHK1^Ser345^ can phosphorylate CDC25C at Ser^216^, which promotes the binding of CDC25C to 14‐3‐3 protein and restraining of this complex in the cytoplasm. Thus, scarcity of pCDC25C^Ser216^ in the nucleus prevents activation of CDK1/Cyclin B1 leading to G2/M arrest.^9^, ^11^ Previously, we have reported that 70% of the VSCC cases in this cohort have high expression of pCDC25C^Ser216^ in the nucleus.^25^ Therefore, it is possible that despite the high expression of pCHK1^Ser345^, pCDC25C^Ser216^ can still activate CDK1/Cyclin B1 complex and trigger G2/M transition. High levels of pCHK1^Ser345^ in the cancer cells might function as a compensatory DNA repair mechanism helping tumor cells to cope with increased levels of DNA damage and replicative stress.^34^ However, we cannot exclude that despite the pCHK1^Ser345^ activation, the downstream targets are not activated and functional which could lead to bypass of G2 arrest. Haruki et al^32^ previously reported existence of a shorter isoform of CHK1 in small cell lung cancer that can interfere with the function of endogenous CHK1 through competitively interacting with endogenous CHK1 molecules.

Compared to the expression of pCHK1^Ser317^, pCHK1^Ser296^, and pCHK1^Ser280^ in normal vulvar squamous epithelium, low nuclear levels of these forms were found in 58%, 64%, and 40% of the VSCC, respectively. Reduced levels of pCHK1^Ser317^ and pCHK1^Ser280^ correlated with aggressive tumor features, including deeper invasion and large tumor diameter. There was also a tendency for a worse prognosis for patients with simultaneously low expression of nuclear pCHK1^Ser317^ and pCHK1^Ser296^. Interestingly, opposing effects were observed for cytoplasmic pCHK1^Ser296^ that correlated with poor differentiation. These results suggest that in addition to differential phosphorylation events, spatiotemporal regulation of CHK1 is crucial for regulation of its functions in the cell cycle in VSCC. As it is established that HPV‐positive VSCC represent an etiological subgroup 2 and our cohort has previously been examined for HPV status,^35^ we analyzed CHK1 activation status separately in these two groups. We found no significant correlation between the phosphorylated forms of CHK1 and survival in either group.

In the nucleus, pCHK1^Ser317^, pCHK1^Ser296^, and pCHK1^Ser280^ proteins were positively correlated with pCDC25C^Ser216^, Wee1, and/or CDK1^Tyr15^/pCyclin B1^Ser126^ which is in line with previous findings showing that these proteins are downstream targets of activated CHK1.^12^ Wee1 inhibits CDK1 by phosphorylating it on Tyr15, and in our cohort, 26% of the VSCC cases show high nuclear expression of Wee1.^24^ For this reason, it is possible that the low nuclear activation of CHK1 results in insufficient regulation of downstream targets, further leading to disruption of checkpoint, increased G2/M transition, and genomic instability that drives tumorigenesis.

Previous studies have suggested that even though the cytoplasmic pool of CHK1 is important for checkpoint function, it is the nuclear pool of CHK1 that supports cell viability,^36^ which does not directly support our observations in VSCC. However, few reports have implicated CHK1 in apoptosis via CHK1‐mediated phosphorylation of p73 at Ser47, resulting in a strong increase in p73 transcription activity in response to DNA damage.^37^ As p73 is overexpressed in vulvar cancer,^38^ one might speculate that the low activation of CHK1 in nucleus impairs p73‐mediated apoptosis and thereby provides cancer cells with survival advantages. Still, neither cytoplasmic nor nuclear expression of any of these phosphorylated forms was significantly associated with disease‐specific survival.

Due to frequent alternations of CHK1 and dependency of cancer cells on G2 checkpoint, targeting of CHK1 is considered as a therapeutic approach in several cancer types. For example, targeting CHK1 significantly enhances cell killing effect by chemotherapy or radiation therapy in ovarian, triple negative breast, and brain cancers.^9^ In vitro targeting of CHK1 by inhibitor or siRNA in vulvar cancer cell lines CAL39 and SW954, both harboring p53 mutations,^39^ leads to a significant reduction in viability which is in line with observation in other cancer types. We observed strong induction of DNA damage and apoptosis. Recently, Kashofer et al^40^ reported that 76% cases of HPV‐negative VSCC harbor p53 mutation which correlates with worse survival, emphasizing the importance of p53 mutation in this VSCC subgroup. For this reason, targeting CHK1 might represent a good therapeutic approach for these patients.

We observed a cell‐specific sensitivity to the CHK1 inhibitor even though the changes of the main downstream CHK1 effectors were comparable between the lines, suggesting that additional unidentified factors are involved. Interestingly, siRNA mediates CHK1 knockout led to stronger induction of DNA damage and apoptosis in SW954 cell line that displayed higher resistance to CHK1 inhibitor. This discrepancy might be due to different mechanism of the targeting. While inhibitor functions by the occupation of the ATP‐binding site of CHK1 with minimal influence on the protein conformation, siRNA targeting removes the protein completely.^19^, ^41^ For this reason, it is possible that despite the presence of inhibitor, CHK1 retains functions independent of ATP‐binding sites.

Taken together, our findings suggest that in VSCC deregulation of CHK1 function occurs and contributes to tumorigenesis. Cellular functions regulated by CHK1 are dependent on CHK1 phosphorylation status and localization. However, none of the phosphorylated CHK1 forms independently correlates to prognosis. Targeting CHK1 in VSCC tumors harboring p53 mutation leads to reduce viability, suggesting that CHK1 might represent good antitumor strategy for this subgroup of tumors.

## CONFLICT OF INTEREST

None of the authors declare conflict of interests.
