# Supplementary material for: Evaluation of CHK1 activation in vulvar squamous cell carcinoma and its potential as a therapeutic target in vitro
Source: Cancer Med. 2018 Jul 2;7(8):3955–64. doi: 10.1002/cam4.1638 (PMC6089182; doi:10.1002/cam4.1638)
Supplement: Supplementary file 1 [file CAM4-7-3955-s001.docx]

**Table S1.** Immunostaining results for pCHK1^Ser345^, pCHK1^Ser317^, pCHK1^Ser296^ and pCHK1^Ser280^

|  |  | **pCHK1^Ser345^** | |  | **pCHK1^Ser317^** | |  | **pCHK1^Ser296^** | |  | **pCHK1^Ser280^** | |
| --- | --- | --- | --- | --- | --- | --- | --- | --- | --- | --- | --- | --- |
| **Score** |  | **C (%)** | **N (%)** |  | **C (%)** | **N (%)** |  | **C (%)** | **N (%)** |  | **C (%)** | **N (%)** |
| 0 |  | 188 (63.9) | 127 (43.2) |  | 22 (7.5) | 2 (0.7) |  | 238 (81.0) | 52 (17.7) |  | 37 (12.6) | 8 (2.7) |
| 1 |  | 0 (0) | 1 (0.3) |  | 3 (1.0) | 0 (0) |  | 0 (0) | 1 (0.3) |  | 2 (0.7) | 0 (0) |
| 2 |  | 16 (5.4) | 35 (11.9) |  | 37 (12.6) | 13 (4.4) |  | 7 (2.4) | 23 (7.8) |  | 39 (13.3) | 12 (4.1) |
| 3 |  | 45 (15.3) | 68 (23.1) |  | 48 (16.3) | 127 (43.2) |  | 35 (11.9) | 113 (38.4) |  | 42 (14.3) | 61 (20.7) |
| 4 |  | 31 (10.5) | 37 (12.6) |  | 39 (13.3) | 28 (9.5) |  | 5 (1.7) | 38 (12.9) |  | 35 (11.9) | 38 (12.9) |
| 6 |  | 14 (4.8) | 22 (7.5) |  | 100 (34.0) | 98 (33.3) |  | 8 (2.7) | 61 (20.7) |  | 106 (36.1) | 121 (41.2) |
| 9 |  | 0 (0) | 4 (1.4) |  | 45 (15.3) | 26 (8.8) |  | 1 (0.3) | 6 (2.0) |  | 33 (11.2) | 54 (18.4) |
| Total |  | 294 (100) | 294 (100) |  | 294 (100) | 294 (100) |  | 294 (100) | 294 (100) |  | 294 (100) | 294 (100) |

C: Cytoplasm

N: Nucleus
