# Supplementary material for: Evaluation of CHK1 activation in vulvar squamous cell carcinoma and its potential as a therapeutic target in vitro
Source: Cancer Med. 2018 Jul 2;7(8):3955–64. doi: 10.1002/cam4.1638 (PMC6089182; doi:10.1002/cam4.1638)
Supplement: Supplementary file 2 [file CAM4-7-3955-s002.doc]

**Table S2.** pCHK1^Ser345^ and pCHK1^Ser317^expression in relation to cell cycle proteins

| **Variables** |  | **pCHK1^Ser345^** | | | | | | |  | **pCHK1^Ser317^** | | | | | | |  |
| --- | --- | --- | --- | --- | --- | --- | --- | --- | --- | --- | --- | --- | --- | --- | --- | --- | --- |
|  |  | **(C)** | | |  | **(N)** | | |  | **(C)** | | |  | **(N)** | | | |
|  | **No.** | **High** | **(%)** | ***p^1^*** |  | **High** | **(%)** | ***p^1^*** |  | **High** | **(%)** | ***p^1^*** |  | **High** | **(%)** | ***p^1^*** | |
| pCHK1^Ser345^ (C) |  |  |  | - |  |  |  | - |  |  |  | <0.001 |  |  |  | 0.058 | |
| Low (≤ 0) | 188 | - | - |  |  | - | - |  |  | 14 | (7) |  |  | 87 | (46) |  | |
| High (> 0) | 106 | - | - |  |  | - | - |  |  | 31 | (29) |  |  | 37 | (35) |  | |
| pCHK1^Ser345^ (N) |  |  |  | - |  |  |  | - |  |  |  | 0.077 |  |  |  | 0.057 | |
| Low (≤ 0) | 128 | - | - |  |  | - | - |  |  | 25 | (20) |  |  | 46 | (36) |  | |
| High (> 0) | 166 | - | - |  |  | - | - |  |  | 30 | (12) |  |  | 78 | (47) |  | |
| pCHK1^Ser317^ (C) |  |  |  | <0.001 |  |  |  | 0.077 |  |  |  | - |  |  |  | - | |
| Low (≤ 6) | 62 | 9 | (15) |  |  | 33 | (53) |  |  | - | - |  |  | - | - |  | |
| High (> 6) | 232 | 97 | (42) |  |  | 133 | (57) |  |  | - | - |  |  | - | - |  | |
| pCHK1^Ser317^ (N) |  |  |  | 0.058 |  |  |  | 0.057 |  |  |  | - |  |  |  | - | |
| Low (≤ 4 ) | 170 | 69 | (41) |  |  | 88 | (52) |  |  | - | - |  |  | - | - |  | |
| High (> 4 ) | 124 | 37 | (30) |  |  | 78 | (63) |  |  | - | - |  |  | - | - |  | |
| pCHK1^Ser296^ (C) |  |  |  | 0.195 |  |  |  | 0.166 |  |  |  | 0.141 |  |  |  | 0.678 | |
| Low (≤ 0) | 238 | 90 | (38) |  |  | 139 | (58) |  |  | 40 | (17) |  |  | 99 | (42) |  | |
| High (> 0) | 56 | 16 | (29) |  |  | 27 | (48) |  |  | 5 | (9) |  |  | 25 | (45) |  | |
| pCHK1^Ser296^ (N) |  |  |  | 0.971 |  |  |  | 0.058 |  |  |  | 0.484 |  |  |  | <0.001 | |
| Low (≤ 3) | 189 | 68 | (36) |  |  | 99 | (52) |  |  | 31 | (16) |  |  | 64 | (34) |  | |
| High (> 3) | 105 | 38 | (36) |  |  | 67 | (64) |  |  | 14 | (13) |  |  | 60 | (57) |  | |
| pCHK1^Ser280^ (C) |  |  |  | <0.001 |  |  |  | 0.077 |  |  |  | <0.001 |  |  |  | 0.183 | |
| Low (≤ 4) | 155 | 40 | (26) |  |  | 80 | (52) |  |  | 10 | (7) |  |  | 71 | (46) |  | |
| High (> 4) | 139 | 66 | (48) |  |  | 86 | (62) |  |  | 35 | (25) |  |  | 53 | (38) |  | |
| pCHK1^Ser280^ (N) |  |  |  | 0.472 |  |  |  | 0.085 |  |  |  | 0.944 |  |  |  | 0.002 | |
| Low (≤ 4) | 119 | 40 | (34) |  |  | 60 | (50) |  |  | 18 | (15) |  |  | 37 | (31) |  | |
| High (> 4) | 175 | 66 | (38) |  |  | 106 | (61) |  |  | 27 | (15) |  |  | 87 | (50) |  | |
| CDK1^Tyr15^ (C) |  |  |  | 0.537 |  |  |  | 0.213 |  |  |  | 0.010 |  |  |  | 0.371 | |
| Low (<3) | 101 | 34 | (34) |  |  | 52 | (52) |  |  | 23 | (23) |  |  | 39 | (39) |  | |
| High (≥3) | 193 | 72 | (37) |  |  | 114 | (59) |  |  | 22 | (11) |  |  | 85 | (44) |  | |
| CDK1^Tyr15^ (N) |  |  |  | 0.012 |  |  |  | 0.430 |  |  |  | 0.070 |  |  |  | 0.007 | |
| Low (<3) | 216 | 87 | (40) |  |  | 119 | (55) |  |  | 38 | (18) |  |  | 81 | (38) |  | |
| High (≥3) | 78 | 19 | (24 |  |  | 47 | (40 |  |  | 7 | (9) |  |  | 43 | (55) |  | |
| pCDK1^Thr161^ (C) |  |  |  | 0.719 |  |  |  | 0.088 |  |  |  | 0.001 |  |  |  | 0.531 | |
| Low (<3) | 198 | 70 | (35) |  |  | 105 | (53) |  |  | 40 | (20) |  |  | 86 | (43) |  | |
| High (≥3) | 96 | 36 | (38) |  |  | 61 | (64) |  |  | 5 | (5) |  |  | 38 | (40) |  | |
| Cyclin B1(Total) (C) |  |  |  | 0.847 |  |  |  | 0.144 |  |  |  | 0.021 |  |  |  | 0.076 | |
| Low (<3) | 88 | 31 | (35) |  |  | 44 | (51) |  |  | 20 | (23) |  |  | 44 | (50) |  | |
| High (≥3) | 206 | 75 | (36) |  |  | 122 | (59) |  |  | 25 | (12) |  |  | 80 | (39) |  | |
| Cyclin B1(Total) (N) |  |  |  | 0.906 |  |  |  | 0.332 |  |  |  | 0.045 |  |  |  | 0.369 | |
| Low (<3) | 212 | 76 | (36) |  |  | 116 | (55) |  |  | 38 | (18) |  |  | 86 | (41) |  | |
| High (≥3) | 82 | 30 | (37) |  |  | 50 | (61) |  |  | 7 | (9) |  |  | 38 | (46) |  | |
| pCyclin B1^Ser126^ (C) |  |  |  | 0.786 |  |  |  | 0.022 |  |  |  | 0.059 |  |  |  | 0.001 | |
| Low (<3) | 222 | 81 | (37) |  |  | 117 | (53) |  |  | 39 | (18) |  |  | 81 | (37) |  | |
| High (≥3) | 72 | 25 | (37) |  |  | 49 | (68) |  |  | 6 | (8) |  |  | 43 | (60) |  | |
| pCyclin B1^Ser126^ (N) |  |  |  | 0.929 |  |  |  | 0.012 |  |  |  | 0.047 |  |  |  | 0.001 | |
| Low (<3) | 220 | 79 | (36) |  |  | 115 | (52) |  |  | 39 | (18) |  |  | 80 | (36) |  | |
| High (≥3) | 74 | 27 | (37) |  |  | 51 | (69) |  |  | 6 | (8) |  |  | 44 | (60) |  | |
| 14-3-3σ (C)^2^ |  |  |  | 0.010 |  |  |  | 0.259 |  |  |  | 0.842 |  |  |  | 0.013 | |
| Low (≤) | 82 | 20 | (24) |  |  | 42 | (51) |  |  | 12 | (15) |  |  | 44 | (54) |  | |
| High (> ) | 212 | 86 | (41) |  |  | 124 | (59) |  |  | 33 | (16) |  |  | 80 | (38) |  | |
| 14-3-3β (C)^2^ |  |  |  | 0.036 |  |  |  | 0.651 |  |  |  | 0.791 |  |  |  | 0.125 | |
| Low (≤1) | 61 | 15 | (25) |  |  | 36 | (59) |  |  | 10 | (16) |  |  | 31 | (51) |  | |
| High (>1) | 233 | 91 | (39) |  |  | 130 | (56) |  |  | 35 | (15) |  |  | 93 | (40) |  | |
| 14-3-3β (N)^2^ |  |  |  | 0.039 |  |  |  | 0.534 |  |  |  | 0.144 |  |  |  | 0.958 | |
| Low (≤ ) | 270 | 102 | (38) |  |  | 151 | (56) |  |  | 44 | (16) |  |  | 114 | (42) |  | |
| High (> ) | 24 | 4 | (17) |  |  | 15 | (63) |  |  | 1 | (4) |  |  | 10 | (42) |  | |
| 14-3-3γ (C)^2^ |  |  |  | 0.362 |  |  |  | 0.636 |  |  |  | 0.050 |  |  |  | 0.109 | |
| Low (≤3) | 124 | 41 | (33) |  |  | 72 | (58) |  |  | 13 | (11) |  |  | 59 | (48) |  | |
| High (>3) | 170 | 65 | (38) |  |  | 94 | (55) |  |  | 32 | (19) |  |  | 65 | (38) |  | |
| 14-3-3ζ (C)^2^ |  |  |  | 0.002 |  |  |  | 0.080 |  |  |  | 0.278 |  |  |  | 0.002 | |
| Low (≤3) | 146 | 40 | (27) |  |  | 75 | (51) |  |  | 19 | (13) |  |  | 75 | (51) |  | |
| High (>3) | 148 | 66 | (45) |  |  | 91 | (62) |  |  | 26 | (18) |  |  | 49 | (33) |  | |
| 14-3-3ζ (N)^2^ |  |  |  | 0.023 |  |  |  | 0.001 |  |  |  | 0.712 |  |  |  | 0.080 | |
| Low (≤6) | 222 | 72 | (32) |  |  | 113 | (51) |  |  | 33 | (15) |  |  | 100 | (45) |  | |
| High (>6) | 72 | 34 | (47) |  |  | 53 | (74) |  |  | 12 | (17) |  |  | 24 | (33) |  | |
| 14-3-3ε (N)^2^ |  |  |  | 0.499 |  |  |  | 0.483 |  |  |  | 0.105 |  |  |  | 0.008 | |
| Low (≤6) | 198 | 74 | (37) |  |  | 109 | (55) |  |  | 35 | (18) |  |  | 73 | (37) |  | |
| High (>6) | 96 | 32 | (33) |  |  | 57 | (59) |  |  | 10 | (10) |  |  | 51 | (53) |  | |
| 14-3-3tau (N)^2^ |  |  |  | 0.385 |  |  |  | 0.909 |  |  |  | 0.316 |  |  |  | 0.001 | |
| Low (≤ ) | 238 | 83 | (35) |  |  | 134 | (56) |  |  | 34 | (14) |  |  | 89 | (37) |  | |
| High (> ) | 56 | 23 | (41) |  |  | 32 | (57) |  |  | 11 | (20) |  |  | 35 | (63) |  | |
| CDC25A (N)^2^ |  |  |  | 0.050 |  |  |  | 0.690 |  |  |  | 0.108 |  |  |  | 0.378 | |
| Low (≤6) | 144 | 60 | (42) |  |  | 83 | (58) |  |  | 27 | (19) |  |  | 57 | (40) |  | |
| High (>6) | 150 | 46 | (31) |  |  | 83 | (55) |  |  | 18 | (12) |  |  | 67 | (45) |  | |
| CDC25B (N)^2^ |  |  |  | 0.125 |  |  |  | 0.327 |  |  |  | 0.025 |  |  |  | 0.032 | |
| Low (≤6) | 248 | 94 | (38) |  |  | 137 | (55) |  |  | 43 | (17) |  |  | 98 | (40) |  | |
| High (>6) | 46 | 12 | (26) |  |  | 29 | (63) |  |  | 2 | (4) |  |  | 26 | (57) |  | |
| CDC25C (C)^2^ |  |  |  | 0.007 |  |  |  | 0.979 |  |  |  | 0.539 |  |  |  | 0.063 | |
| Low (≤3) | 110 | 29 | (26) |  |  | 62 | (56) |  |  | 15 | (14) |  |  | 54 | (49) |  | |
| High (>3) | 184 | 77 | (42) |  |  | 104 | (57) |  |  | 30 | (16) |  |  | 70 | (38) |  | |
| pCDC25C^Ser216^ (C)^2^ |  |  |  | 0.061 |  |  |  | 0.499 |  |  |  | 0.004 |  |  |  | 0.971 | |
| Low (≤3) | 145 | 60 | (41) |  |  | 79 | (55) |  |  | 31 | (21) |  |  | 61 | (42) |  | |
| High (>3) | 149 | 46 | (31) |  |  | 87 | (58) |  |  | 14 | (9) |  |  | 63 | (42) |  | |
| pCDC25C^Ser216^ (N)^2^ |  |  |  | <0.001 |  |  |  | <0.001 |  |  |  | 0.954 |  |  |  | 0.005 | |
| Low (≤0) | 86 | 15 | (17) |  |  | 31 | (36) |  |  | 13 | (15) |  |  | 47 | (55) |  | |
| High (>0) | 208 | 91 | (44) |  |  | 135 | (65) |  |  | 32 | (15) |  |  | 77 | (37) |  | |
| Wee1 (N)^2^ |  |  |  | 0.020 |  |  |  | 0.770 |  |  |  | 0.815 |  |  |  | 0.287 | |
| Low (≤6) | 218 | 87 | (40) |  |  | 122 | (56) |  |  | 34 | (16) |  |  | 88 | (40) |  | |
| High (>6) | 76 | 19 | (25) |  |  | 44 | (58) |  |  | 11 | (15) |  |  | 36 | (47) |  | |
| Hpv^2^ |  |  |  | 0.011 |  |  |  | 0.234 |  |  |  | 0.083 |  |  |  | 0.002 | |
| Low (-) | 164 | 73 | (45) |  |  | 95 | (58) |  |  | 136 | (83) |  |  | 62 | (38) |  | |
| High (+) | 40 | 9 | (23) |  |  | 19 | (48) |  |  | 28 | (70) |  |  | 26 | (65) |  | |
| Not available | 90 |  |  |  |  |  |  |  |  |  |  |  |  |  |  |  | |

^1^Pearson chi-square

^2^Factors reported in previous studies (24-28)

C: Cytoplasm

N: Nucleus

pCHK1^Ser345^ C High and N High: Immunostaining score > 0; pCHK1^Ser317^ C High: Immunostaining score > 6, N High: Immunostaining score > 4
