# Supplementary material for: Evaluation of CHK1 activation in vulvar squamous cell carcinoma and its potential as a therapeutic target in vitro
Source: Cancer Med. 2018 Jul 2;7(8):3955–64. doi: 10.1002/cam4.1638 (PMC6089182; doi:10.1002/cam4.1638)
Supplement: Supplementary file 3 [file CAM4-7-3955-s003.doc]

**Table S3.** pCHK1^Ser296^ and pCHK1^Ser280^expression in relation to cell cycle proteins

| **Variables** |  | **pCHK1^Ser296^** | | | | | | |  | **pCHK1^Ser280^** | | | | | | |
| --- | --- | --- | --- | --- | --- | --- | --- | --- | --- | --- | --- | --- | --- | --- | --- | --- |
|  |  | **(C)** | | |  | **(N)** | | |  | **(C)** | | |  | **(N)** | | |
|  | **No.** | **High** | **(%)** | ***p^1^*** |  | **High** | **(%)** | ***p^1^*** |  | **High** | **(%)** | ***p^1^*** |  | **High** | **(%)** | ***p^1^*** |
| pCHK1^Ser296^ (C) |  |  |  | - |  |  |  | - |  |  |  | 0.661 |  |  |  | 0.480 |
| Low (≤ 0) | 238 | - | - |  |  | - | - |  |  | 114 | (48) |  |  | 144 | (61) |  |
| High (> 3) | 56 | - | - |  |  | - | - |  |  | 25 | (45) |  |  | 31 | (55) |  |
| pCHK1^Ser296^ (N) |  |  |  | - |  |  |  | - |  |  |  | 0.741 |  |  |  | 0.107 |
| Low (≤ 3) | 189 | - | - |  |  | - | - |  |  | 88 | (47) |  |  | 106 | (56) |  |
| High (> 3) | 105 | - | - |  |  | - | - |  |  | 51 | (49) |  |  | 89 | (66) |  |
| pCHK1^Ser280^ (C) |  |  |  | 0.661 |  |  |  | 0.741 |  |  |  | - |  |  |  | - |
| Low (≤ 4) | 155 | 31 | (20) |  |  | 54 | (35) |  |  | - | - |  |  | - | - |  |
| High (> 4) | 139 | 25 | (18) |  |  | 51 | (37) |  |  | - | - |  |  | - | - |  |
| pCHK1^Ser280^ (N) |  |  |  | 0.480 |  |  |  | 0.107 |  |  |  | - |  |  |  | - |
| Low (≤ 4) | 119 | 25 | (21) |  |  | 36 | (30) |  |  | - | - |  |  | - | - |  |
| High (> 4) | 175 | 31 | (17) |  |  | 69 | (39) |  |  | - | - |  |  | - | - |  |
| CDK1^Tyr15^ (C) |  |  |  | 0.311 |  |  |  | <0.001 |  |  |  | 0.242 |  |  |  | 0.638 |
| Low (<3) | 101 | 16 | (16) |  |  | 18 | (18) |  |  | 43 | (43) |  |  | 62 | (61) |  |
| High (≥3) | 193 | 40 | (21) |  |  | 87 | (45) |  |  | 96 | (50) |  |  | 113 | (59) |  |
| CDK1^Tyr15^ (N) |  |  |  | 0.084 |  |  |  | <0.001 |  |  |  | 0.816 |  |  |  | 0.134 |
| Low (<3) | 216 | 36 | (17) |  |  | 60 | (28) |  |  | 103 | (48) |  |  | 123 | (57) |  |
| High (≥3) | 78 | 20 | (26) |  |  | 45 | (58) |  |  | 36 | (46) |  |  | 52 | (67) |  |
| pCDK1^Thr161^ (C) |  |  |  | 0.015 |  |  |  | 0.081 |  |  |  | 0.066 |  |  |  | 0.070 |
| Low (<3) | 198 | 30 | (15) |  |  | 64 | (33) |  |  | 101 | (51) |  |  | 125 | (63) |  |
| High (≥3) | 96 | 26 | (27) |  |  | 41 | (43) |  |  | 38 | (40) |  |  | 50 | (52) |  |
| pCDK1^Thr161^ (N) |  |  |  | 0.104 |  |  |  | 0.005 |  |  |  | 0.288 |  |  |  | 0.935 |
| Low (<3) | 66 | 8 | (12) |  |  | 14 | (21) |  |  | 35 | (53) |  |  | 39 | (59) |  |
| High (≥3) | 228 | 48 | (21) |  |  | 91 | (40) |  |  | 104 | (46) |  |  | 136 | (60) |  |
| Cyclin B1(Total) (C) |  |  |  | 0.123 |  |  |  | 0.006 |  |  |  | 0.153 |  |  |  | 0.048 |
| Low (<3) | 88 | 12 | (14) |  |  | 21 | (24) |  |  | 36 | (41) |  |  | 60 | (68) |  |
| High (≥3) | 206 | 44 | (21) |  |  | 84 | (41) |  |  | 103 | (50) |  |  | 115 | (56) |  |
| Cyclin B1(Total) (N) |  |  |  | 0.075 |  |  |  | 0.008 |  |  |  | 0.400 |  |  |  | 0.632 |
| Low (<3) | 212 | 35 | (17) |  |  | 66 | (31) |  |  | 97 | (46) |  |  | 128 | (60) |  |
| High (≥3) | 82 | 21 | (26) |  |  | 39 | (48) |  |  | 42 | (51) |  |  | 47 | (57) |  |
| pCyclin B1^Ser126^ (C) |  |  |  | 0.430 |  |  |  | <0.001 |  |  |  | 0.777 |  |  |  | 0.155 |
| Low (<3) | 222 | 40 | (18) |  |  | 62 | (29) |  |  | 106 | (48) |  |  | 127 | (57) |  |
| High (≥3) | 72 | 16 | (22) |  |  | 43 | (60) |  |  | 33 | (46) |  |  | 48 | (67) |  |
| pCyclin B1^Ser126^ (N) |  |  |  | 0.320 |  |  |  | <0.001 |  |  |  | 0.997 |  |  |  | 0.175 |
| Low (<3) | 220 | 39 | (18) |  |  | 62 | (28) |  |  | 104 | (47) |  |  | 126 | (57) |  |
| High (≥3) | 74 | 17 | (23) |  |  | 43 | (58) |  |  | 35 | (47) |  |  | 49 | (66) |  |
| 14-3-3σ (C)^2^ |  |  |  | 0.231 |  |  |  | 0.048 |  |  |  | 0.011 |  |  |  | 0.398 |
| Low (≤ ) | 82 | 12 | (15) |  |  | 22 | (27) |  |  | 29 | (35) |  |  | 52 | (63) |  |
| High (> ) | 212 | 44 | (21) |  |  | 83 | (39) |  |  | 110 | (52) |  |  | 123 | (58) |  |
| 14-3-3β (C)^2^ |  |  |  | 0.185 |  |  |  | 0.506 |  |  |  | 0.049 |  |  |  | 0.840 |
| Low (≤1) | 61 | 8 | (13) |  |  | 24 | (39) |  |  | 22 | (36) |  |  | 37 | (61) |  |
| High (>1) | 233 | 48 | (21) |  |  | 81 | (35) |  |  | 117 | (50) |  |  | 138 | (59) |  |
| 14-3-3γ (C)^2^ |  |  |  | 0.165 |  |  |  | 0.751 |  |  |  | 0.023 |  |  |  | 0.084 |
| Low (≤3) | 124 | 19 | (15) |  |  | 43 | (35) |  |  | 49 | (40) |  |  | 81 | (65) |  |
| High (>3) | 170 | 37 | (22) |  |  | 62 | (37) |  |  | 90 | (53) |  |  | 94 | (55) |  |
| 14-3-3ζ (C)^2^ |  |  |  | 0.810 |  |  |  | 0.444 |  |  |  | 0.010 |  |  |  | 0.147 |
| Low (≤3) | 146 | 27 | (19) |  |  | 49 | (34) |  |  | 58 | (40) |  |  | 93 | (64) |  |
| High (>3) | 148 | 29 | (20) |  |  | 56 | (38) |  |  | 81 | (55) |  |  | 82 | (55) |  |
| 14-3-3ζ (N)^2^ |  |  |  | 0.805 |  |  |  | 0.293 |  |  |  | 0.595 |  |  |  | 0.012 |
| Low (≤6) | 222 | 43 | (19) |  |  | 83 | (37) |  |  | 103 | (46) |  |  | 123 | (55) |  |
| High (>6) | 72 | 13 | (18) |  |  | 22 | (31) |  |  | 36 | (50) |  |  | 52 | (72) |  |
| 14-3-3η (C)^2^ |  |  |  | 0.002 |  |  |  | 0.024 |  |  |  | 0.320 |  |  |  | 0.358 |
| Low (≤3) | 138 | 16 | (12) |  |  | 40 | (29) |  |  | 61 | (44) |  |  | 86 | (62) |  |
| High (>3) | 156 | 40 | (26) |  |  | 65 | (42) |  |  | 78 | (50) |  |  | 89 | (57) |  |
| 14-3-3ε (C)^2^ |  |  |  | 0.729 |  |  |  | 0.201 |  |  |  | 0.031 |  |  |  | 0.584 |
| Low (≤1) | 41 | 7 | (17) |  |  | 11 | (27) |  |  | 13 | (32) |  |  | 26 | (63) |  |
| High (>1) | 253 | 49 | (19) |  |  | 94 | (37) |  |  | 126 | (50) |  |  | 149 | (59) |  |
| 14-3-3ε (N)^2^ |  |  |  | 0.469 |  |  |  | 0.081 |  |  |  | 0.399 |  |  |  | 0.025 |
| Low (≤6) | 198 | 40 | (20) |  |  | 64 | (32) |  |  | 97 | (49) |  |  | 109 | (55) |  |
| High (>6) | 96 | 16 | (17) |  |  | 41 | (43) |  |  | 42 | (44) |  |  | 66 | (69) |  |
| 14-3-3tau (N)^2^ |  |  |  | 0.900 |  |  |  | 0.005 |  |  |  | 0.301 |  |  |  | 0.840 |
| Low (≤ 4 ) | 238 | 45 | (19) |  |  | 76 | (32) |  |  | 116 | (49) |  |  | 141 | (59) |  |
| High (> 4) | 56 | 11 | (20) |  |  | 29 | (52) |  |  | 23 | (41) |  |  | 34 | (61) |  |
| CDC25C (C)^2^ |  |  |  | 0.549 |  |  |  | 0.091 |  |  |  | 0.999 |  |  |  | 0.019 |
| Low (≤3) | 110 | 19 | (17) |  |  | 46 | (42) |  |  | 52 | (47) |  |  | 75 | (68) |  |
| High (>3) | 184 | 37 | (20) |  |  | 59 | (32) |  |  | 87 | (47) |  |  | 100 | (54) |  |
| pCDC25C^Ser216^ (C)^2^ |  |  |  | 0.024 |  |  |  | 0.002 |  |  |  | 0.917 |  |  |  | 0.941 |
| Low (≤3) | 145 | 20 | (14) |  |  | 39 | (27) |  |  | 69 | (48) |  |  | 86 | (59) |  |
| High (>3) | 149 | 36 | (24) |  |  | 66 | (44) |  |  | 70 | (47) |  |  | 89 | (60) |  |
| pCDC25C^Ser216^ (N)^2^ |  |  |  | 0.237 |  |  |  | 0.001 |  |  |  | 0.049 |  |  |  | 0.960 |
| Low (≤0) | 86 | 20 | (23) |  |  | 43 | (50) |  |  | 33 | (38) |  |  | 51 | (59) |  |
| High (>0) | 208 | 36 | (17) |  |  | 62 | (30) |  |  | 106 | (51) |  |  | 124 | (60) |  |
| Wee1 (C)^2^ |  |  |  | 0.496 |  |  |  | 0.043 |  |  |  | 0.860 |  |  |  | 0.838 |
| Low (≤0) | 138 | 24 | (17) |  |  | 41 | (30) |  |  | 66 | (48) |  |  | 83 | (60) |  |
| High (>0) | 156 | 32 | (21) |  |  | 64 | (41) |  |  | 73 | (47) |  |  | 92 | (59) |  |
| Wee1 (N)^2^ |  |  |  | 0.125 |  |  |  | <0.001 |  |  |  | 0.188 |  |  |  | 0.948 |
| Low (≤6) | 218 | 37 | (17) |  |  | 61 | (28) |  |  | 108 | (50) |  |  | 130 | (60) |  |
| High (>6) | 76 | 19 | (25) |  |  | 44 | (58) |  |  | 31 | (41) |  |  | 45 | (59) |  |
| Hpv^2^ |  |  |  | 0.388 |  |  |  | 0.006 |  |  |  | 0.439 |  |  |  | 0.910 |
| Low (-) | 164 | 31 | (19) |  |  | 45 | (27) |  |  | 85 | (52) |  |  | 100 | (61) |  |
| High (+) | 40 | 10 | (25) |  |  | 20 | (50) |  |  | 18 | (45) |  |  | 24 | (60) |  |
| Not available | 90 |  |  |  |  |  |  |  |  |  |  |  |  |  |  |  |

^1^Pearson chi-square

^2^Factors reported in previous studies (24-28)

C: Cytoplasm

N: Nucleus

pCHK1^Ser296^ C High: Immunostaining score > 0, N High: Immunostaining score > 3; pCHK1^Ser280^ C High and N High: Immunostaining score > 4
