# Supplementary material for: Evaluation of CHK1 activation in vulvar squamous cell carcinoma and its potential as a therapeutic target in vitro
Source: Cancer Med. 2018 Jul 2;7(8):3955–64. doi: 10.1002/cam4.1638 (PMC6089182; doi:10.1002/cam4.1638)
Supplement: Supplementary file 4 [file CAM4-7-3955-s004.docx]

**Table S4.** pCHK1^Ser345^ and pCHK1^Ser317^ expression in relation to clinicopathological variables

|  |  | **pCHK1^Ser345^** | | | | | | |  | **pCHK1^Ser317^** | | | | | | |
| --- | --- | --- | --- | --- | --- | --- | --- | --- | --- | --- | --- | --- | --- | --- | --- | --- |
| **Variables** |  | **(C)** | | |  | **(N)** | | |  | **(C)** | | |  | **(N)** | | |
|  | **No.** | **High** | **(%)** | ***p*** |  | **High** | **(%)** | ***p*** |  | **High** | **(%)** | ***p*** |  | **High** | **(%)** | ***p*** |
| Age |  |  |  | 0.235^1^ |  |  |  | 0.957^1^ |  |  |  | 0.475^1^ |  |  |  | 0.002^1^ |
| 25-69 | 116 | 46 | (40) |  |  | 67 | (58) |  |  | 14 | (12) |  |  | 64 | (55) |  |
| 70-84 | 144 | 50 | (35) |  |  | 78 | (54) |  |  | 26 | (18) |  |  | 48 | (33) |  |
| 85+ | 34 | 10 | (29) |  |  | 21 | (62) |  |  | 5 | (15) |  |  | 12 | (35) |  |
| FIGO |  |  |  | 0.930^2^ |  |  |  | 0.340^2^ |  |  |  | 0.445^2^ |  |  |  | 0.686^2^ |
| Ia | 10 | 2 | (20) |  |  | 5 | (50) |  |  | 1 | (10) |  |  | 4 | (40) |  |
| Ib | 136 | 54 | (40) |  |  | 76 | (56) |  |  | 25 | (18) |  |  | 59 | (43) |  |
| II | 13 | 4 | (31) |  |  | 4 | (31) |  |  | 2 | (15) |  |  | 7 | (54) |  |
| IIIa | 62 | 21 | (34) |  |  | 34 | (55) |  |  | 7 | (11) |  |  | 28 | (45) |  |
| IIIb | 38 | 14 | (37) |  |  | 24 | (63) |  |  | 6 | (16) |  |  | 11 | (29) |  |
| IIIc | 12 | 4 | (33) |  |  | 6 | (50) |  |  | 2 | (17) |  |  | 4 | (33) |  |
| IVa | 5 | 1 | (20) |  |  | 4 | (80) |  |  | 2 | (40) |  |  | 3 | (60) |  |
| IVb | 13 | 4 | (31) |  |  | 10 | (77) |  |  | 0 | (0) |  |  | 6 | (46) |  |
| Not available | 5 |  |  |  |  |  |  |  |  |  |  |  |  |  |  |  |
| Lymph node metastasis |  |  |  | 0.705^3^ |  |  |  | 0.367^3^ |  |  |  | 0.424^3^ |  |  |  | 0.203^3^ |
| None | 163 | 61 | (37) |  |  | 87 | (53) |  |  | 29 | (18) |  |  | 73 | (45) |  |
| Unilateral | 87 | 28 | (32) |  |  | 50 | (58) |  |  | 12 | (14) |  |  | 37 | (43) |  |
| Bilateral | 38 | 14 | (37) |  |  | 25 | (66) |  |  | 4 | (11) |  |  | 11 | (29) |  |
| Not available | 6 |  |  |  |  |  |  |  |  |  |  |  |  |  |  |  |
| Tumor diameter (cm) |  |  |  | 0.932^1^ |  |  |  | 0.937^1^ |  |  |  | 0.141^1^ |  |  |  | 0.001^1^ |
| 0.3-2.5 | 85 | 33 | (39) |  |  | 45 | (53) |  |  | 15 | (18) |  |  | 48 | (57) |  |
| 2.6-4.0 | 93 | 32 | (34) |  |  | 58 | (62) |  |  | 12 | (13) |  |  | 35 | (38) |  |
| 4.1-20.0 | 100 | 38 | (38) |  |  | 54 | (54) |  |  | 14 | (14) |  |  | 32 | (32) |  |
| Not available | 16 |  |  |  |  |  |  |  |  |  |  |  |  |  |  |  |
| Tumor differentiation |  |  |  | 0.298^3^ |  |  |  | 0.729^3^ |  |  |  | 0.093^3^ |  |  |  | 0.625^3^ |
| Well | 73 | 31 | (43) |  |  | 39 | (53) |  |  | 17 | (23) |  |  | 30 | (41) |  |
| Moderate | 151 | 54 | (36) |  |  | 85 | (56) |  |  | 21 | (14) |  |  | 61 | (40) |  |
| Poor | 70 | 21 | (30) |  |  | 42 | (60) |  |  | 7 | (10) |  |  | 33 | (47) |  |
| Depth of invasion (mm) |  |  |  | 0.275^1^ |  |  |  | 0.219^1^ |  |  |  | 0.486^1^ |  |  |  | <0.001^1^ |
| 0.0-4.0 | 74 | 31 | (42) |  |  | 43 | (58) |  |  | 8 | (11) |  |  | 46 | (62) |  |
| 4.1-8.0 | 97 | 36 | (37) |  |  | 59 | (61) |  |  | 20 | (21) |  |  | 39 | (40) |  |
| 8.1-40.0 | 112 | 38 | (34) |  |  | 56 | (50) |  |  | 15 | (13) |  |  | 31 | (28) |  |
| Not available | 11 |  |  |  |  |  |  |  |  |  |  |  |  |  |  |  |
| Infiltration of vessel |  |  |  | 0.946^3^ |  |  |  | 0.967^3^ |  |  |  | 0.829^3^ |  |  |  | 0.881^3^ |
| No | 226 | 81 | (36) |  |  | 128 | (57) |  |  | 35 | (16) |  |  | 95 | (42) |  |
| Yes | 65 | 23 | (35) |  |  | 37 | (57) |  |  | 9 | (14) |  |  | 28 | (43) |  |
| Not available | 3 |  |  |  |  |  |  |  |  |  |  |  |  |  |  |  |

C: Cytoplasm

N: Nucleus

pCHK1^Ser345^ C High and N High: Immunostaining score > 0; pCHK1^Ser317^  C High: Immunostaining score > 6, N High: Immunostaining score > 4

^1^Linear-by-linear association

^2^Fisher exact test

^3^Pearson chi-square
