# Supplementary material for: Evaluation of CHK1 activation in vulvar squamous cell carcinoma and its potential as a therapeutic target in vitro
Source: Cancer Med. 2018 Jul 2;7(8):3955–64. doi: 10.1002/cam4.1638 (PMC6089182; doi:10.1002/cam4.1638)
Supplement: Supplementary file 5 [file CAM4-7-3955-s005.docx]

**Table S5.** pCHK1^Ser296^ and pCHK1^Ser280^ expression in relation to clinicopathological variables

| **Variables** |  | **pCHK1^Ser296^** | | | | | | | |  | **pCHK1^Ser280^** | | | | | | |
| --- | --- | --- | --- | --- | --- | --- | --- | --- | --- | --- | --- | --- | --- | --- | --- | --- | --- |
|  |  | **(C)** | | |  | | **(N)** | | |  | **(C)** | | |  | **(N)** | | |
|  | **No.** | **High** | **(%)** | ***p*** |  | **High** | | **(%)** | ***p*** |  | **High** | **(%)** | ***p*** |  | **High** | **(%)** | ***p*** |
| Age |  |  |  | 0.591^1^ |  |  | |  | 0.030^1^ |  |  |  | 0.967^1^ |  |  |  | 0.492^1^ |
| 25-69 | 116 | 24 | (21) |  |  | 50 | | (43) |  |  | 54 | (47) |  |  | 70 | (60) |  |
| 70-84 | 144 | 26 | (18) |  |  | 46 | | (32) |  |  | 70 | (49) |  |  | 80 | (56) |  |
| 85+ | 34 | 6 | (18) |  |  | 9 | | (27) |  |  | 15 | (44) |  |  | 25 | (74) |  |
| FIGO |  |  |  | 0.435^2^ |  |  | |  | 0.240^2^ |  |  |  | 0.245^2^ |  |  |  | 0.327^2^ |
| Ia | 10 | 1 | (10) |  |  | 4 | | (40) |  |  | 3 | (30) |  |  | 8 | (80) |  |
| Ib | 136 | 24 | (18) |  |  | 46 | | (34) |  |  | 64 | (47) |  |  | 82 | (60) |  |
| II | 13 | 3 | (23) |  |  | 4 | | (31) |  |  | 4 | (31) |  |  | 7 | (54) |  |
| IIIa | 62 | 16 | (26) |  |  | 28 | | (45) |  |  | 27 | (44) |  |  | 31 | (50) |  |
| IIIb | 38 | 9 | (24) |  |  | 11 | | (29) |  |  | 24 | (63) |  |  | 24 | (63) |  |
| IIIc | 12 | 0 | ( 0) |  |  | 2 | | (17) |  |  | 8 | (67) |  |  | 10 | (83) |  |
| IVa | 5 | 0 | ( 0) |  |  | 2 | | (40) |  |  | 2 | (40) |  |  | 2 | (40) |  |
| IVb | 13 | 3 | (23) |  |  | 8 | | (62) |  |  | 5 | (39) |  |  | 8 | (62) |  |
| Not available | 5 |  |  |  |  |  | |  |  |  |  |  |  |  |  |  |  |
| Lymph node metastasis |  |  |  | 0.535^3^ |  |  | |  | 0.163^3^ |  |  |  | 0.304^3^ |  |  |  | 0.467^3^ |
| None | 163 | 28 | (17) |  |  | 54 | | (33) |  |  | 72 | (44) |  |  | 100 | (61) |  |
| Unilateral | 87 | 20 | (23) |  |  | 38 | | (44) |  |  | 42 | (48) |  |  | 47 | (54) |  |
| Bilateral | 38 | 7 | (18) |  |  | 11 | | (29) |  |  | 22 | (58) |  |  | 24 | (63) |  |
| Not available | 6 |  |  |  |  |  | |  |  |  |  |  |  |  |  |  |  |
| Tumor diameter (cm) |  |  |  | 0.831^1^ |  |  | |  | 0.991^1^ |  |  |  | 0.689^1^ |  |  |  | 0.670^1^ |
| 0.3-2.5 | 85 | 15 | (18) |  |  | 32 | | (38) |  |  | 43 | (51) |  |  | 54 | (64) |  |
| 2.6-4.0 | 93 | 19 | (20) |  |  | 25 | | (27) |  |  | 39 | (42) |  |  | 50 | (54) |  |
| 4.1-20.0 | 100 | 19 | (19) |  |  | 37 | | (37) |  |  | 53 | (53) |  |  | 60 | (60) |  |
| Not available | 16 |  |  |  |  |  | |  |  |  |  |  |  |  |  |  |  |
| Tumor differentiation |  |  |  | 0.025^3^ |  |  | |  | 0.056^3^ |  |  |  | 0.034^3^ |  |  |  | 0.758^3^ |
| Well | 73 | 10 | (14) |  |  | 18 | | (25) |  |  | 40 | (55) |  |  | 46 | (63) |  |
| Moderate | 151 | 25 | (17) |  |  | 62 | | (41) |  |  | 75 | (50) |  |  | 89 | (59) |  |
| Poor | 70 | 21 | (30) |  |  | 25 | | (36) |  |  | 24 | (34) |  |  | 40 | (57) |  |
| Depth of invasion (mm) |  |  |  | 0.604^1^ |  |  | |  | 0.529^1^ |  |  |  | 0.405^1^ |  |  |  | 0.019^1^ |
| 0.0-4.0 | 74 | 13 | (18) |  |  | 29 | | (39) |  |  | 34 | (46) |  |  | 58 | (78) |  |
| 4.1-8.0 | 97 | 18 | (19) |  |  | 30 | | (31) |  |  | 45 | (46) |  |  | 45 | (46) |  |
| 8.1-40.0 | 112 | 23 | (21) |  |  | 38 | | (34) |  |  | 58 | (52) |  |  | 65 | (58) |  |
| Not available | 11 |  |  |  |  |  | |  |  |  |  |  |  |  |  |  |  |
| Infiltration of vessel |  |  |  | 0.856^3^ |  |  | |  | 0.603^3^ |  |  |  | 0.215^3^ |  |  |  | 0.540^3^ |
| No | 226 | 44 | (20) |  |  | 79 | | (35) |  |  | 102 | (45) |  |  | 133 | (59) |  |
| Yes | 65 | 12 | (19) |  |  | 25 | | (39) |  |  | 35 | (54) |  |  | 41 | (63) |  |
| Not available | 3 |  |  |  |  |  | |  |  |  |  |  |  |  |  |  |  |

C: Cytoplasm

N: Nucleus

pCHK1^Ser296^ C High: Immunostaining score > 0, N High: Immunostaining score > 3; pCHK1^Ser280^ C High and N High: Immunostaining score > 4

^1^Linear-by-linear association

^2^Fisher exact test

^3^Pearson chi-square
